# The Effect of Plant Tissue and Vaccine Formulation on the Oral Immunogenicity of a Model Plant-Made Antigen in Sheep

**DOI:** 10.1371/journal.pone.0052907

**Published:** 2012-12-20

**Authors:** Assunta Pelosi, David Piedrafita, Giorgio De Guzman, Robert Shepherd, John D. Hamill, Els Meeusen, Amanda M. Walmsley

**Affiliations:** 1 School of Biological Sciences, Monash University, Clayton, Victoria, Australia; 2 Department of Physiology, Monash University, Clayton, Victoria, Australia; TGen, United States of America

## Abstract

Antigen-specific antibody responses against a model antigen (the B subunit of the heat labile toxin of enterotoxigenic *Escherichia coli*, LTB) were studied in sheep following oral immunisation with plant-made and delivered vaccines. Delivery from a root-based vehicle resulted in antigen-specific immune responses in mucosal secretions of the abomasum and small intestine and mesenteric lymph nodes. Immune responses from the corresponding leaf-based vaccine were more robust and included stimulation of antigen-specific antibodies in mucosal secretions of the abomasum. These findings suggest that oral delivery of a plant bioencapsulated antigen can survive passage through the rumen to elicit mucosal and systemic immune responses in sheep. Moreover, the plant tissue used as the vaccine delivery vehicle affects the magnitude of these responses.

## Introduction

Vaccines administered via mucosal routes are sought-after because they can induce both mucosal and systemic immune responses to protect against infections caused by pathogens entering and colonising mucosal surfaces such as the gastrointestinal tract (GIT). Mucosal, humoral responses are characterised by secretory antibodies of which the IgA isotype is the most prominent and IgG less abundant [1], [2]. An effective mucosal vaccine must deliver antigen to mucosal inductive sites including the mucosal lymphoid tissue (MALT) or sub-epithelial dendritic cells (DCs) when MALT is absent [1], [2]. Activated DCs then transport the antigen via the lymphatics to draining mesenteric lymph nodes (MLN) where antigen is presented and a specific immune response mounted. Unfortunately, mucosal immune responses are often variable, particularly when vaccines are delivered orally, exposing the antigen to likely enzymatic degradation in the acidic gastric environment [3]. Vaccine delivery from plant tissues may overcome or at the very least mitigate the hostile gastric environment. Evidence points to antigens bioencapsulated within a plant cell being better protected from the enzymatic degradation of the GIT, prolonging release and presentation of the intact antigen to immune responsive sites of the gut associated lymphoid tissues (GALT) [3]. In addition, plant-made vaccines have a reduced risk of contamination with animal pathogens [4], [5] and are stable at room temperature when stored as seed or freeze-dried material thus reducing the reliance for a cold chain [6], [7].

The heat labile toxin (LT) of enterotoxigenic *Escherichia coli* is a well characterised, mucosal antigen often used as an adjuvant [8], [9] or carrier protein [10]. LT comprises a single, active ADP-ribosylation subunit (LTA) and a non-toxic, pentameric subunit (LTB) [11], [12] that selectively binds GM1 ganglioside receptors in the mucosal epithelium of the GIT [13], [14]. LTB is stable in the hostile environment of the GIT [15], can be produced in transgenic plants and elicits potent antigen-specific immune responses when delivered orally from various plant tissues [3], [10], [16], [17], [18], [19], [20]. As such, LTB was chosen as a model antigen to study immunogenicity of orally delivered plant-made vaccines in ruminant species.

In an earlier study we examined different plant tissues as potential vehicles for oral delivery of recombinant LTB (rLTB) in the mouse GIT [3]. Our findings indicated that the plant tissue type used as the vaccine delivery vehicle affected the timing of antigen release, occurring earlier when delivered from leaf whilst being delayed from root [3]. In this same study, the orally delivered plant-made vaccines produced more robust immune responses when formulated in a lipid (oil) based, rather than an aqueous based medium [3]. On the basis of these preliminary studies in mice, the aim of the present study was to determine whether orally delivered plant-made vaccines survive passage through the more complex ruminant digestive system and induce immune responses in sheep. Leaf- and root-based LTB vaccines, each formulated in a lipid matrix, were compared and antigen-specific antibody responses localised to specific sites in the sheep GIT and mucosal immune system.

## Materials and Methods

### Plant materials

Hairy root cultures of transgenic *Petunia parodii* (petunia) plants producing rLTB were generated and maintained as described previously [3], [21]. Control petunia hairy root cultures were stably transformed with the pBinPlus empty vector [21], [22]. For vaccine batch processing, hairy root cultures were harvested 22 days after subculture, snap frozen in liquid N_2_ then freeze-dried using a Dynavac freeze drier (Model FD12) for 48 h with a maximum shelf temperature of 20°C. *Nicotiana benthamiana* leaves transiently expressing apoplast-targeted LTB or GFP were produced as described previously [3]. Leaves were harvested at 7–10 days post-infiltration, snap-frozen in liquid N_2_ then freeze-dried using a Dynavac freeze drier (Model FD12) for 48 h with a maximum shelf temperature of 20°C. Freeze-dried plant materials were powdered using a commercial coffee grinder and sieved to standardise particle size to 0.5–1 mm^2^. Accumulation of rLTB pentamer, the functional form required for binding to GM1-gangliosides on the mucosal surface of the gut epithelium, was confirmed in *N. bethamiana* leaves and petunia hairy roots as per [3]. In each case, the hairy root and leaf vaccine batches accumulated 300 µg/g dwt rLTB.

### Capture enzyme-linked immunosorbent assay (ELISA) to determine rLTB in vaccine batches

Crude protein was extracted from freeze-dried plant material by homogenising in 1∶60 (w/v) PBST [PBS (137 mM NaCl, 2.7 mM KCl, 10 mM Na_2_HPO_4_, 2 mM KH_2_PO_4_) supplemented with 0.05% Tween 20] with two 3 mm tungsten carbide beads for 1 min at a frequency of 28/s in a Qiagen Mixer Mill. The homogenate was cleared by centrifugation at 13,000 rpm at 4°C for 5 min.

LTB-specific capture ELISA was performed using Costar 9018 96-well microtitre plates (Corning Life Sciences) coated with 50 µl/well of chicken anti-cholera enterotoxin subunit B (CTB) antibody (Sigma-Aldrich) diluted 1∶5,000 in PBS. Plates were sealed and incubated at 4°C overnight. Unless stated otherwise, all subsequent incubations were performed at 37°C for 1 h and antibodies diluted in 1% dry skim milk powder (DM) in PBST. Following all incubations, plates were washed three times with PBST.

Plates were blocked with 5% DM in PBST before a 2 h room temperature (22–25°C) incubation with serially diluted crude plant extract starting with 1∶100 in PBS. Plates were then incubated with 1∶2,000 rabbit anti-LTB (Benchmark Biolabs), then 1∶15,000 goat anti-rabbit IgG HRP conjugate (Sigma-Aldrich). Bound LTB-specific antibodies were visualised using TMB-peroxidase substrate (Bio-Rad Laboratories) according to manufacturer's instructions. The amount of rLTB in the freeze-dried plant materials was calculated against a *Pichia pastoris*-made rLTB (Sigma-Aldrich) standard. Accumulation of the functional pentameric form of rLTB was confirmed by western blot [3].

### Mucosal vaccination of sheep

Outbred, male sheep (*Ovis aries*, Merion/Merino) aged between 4.5 to 12 months were obtained from the Commercial Registered Pfizer Animal Health Woodend Farm and housed at the Monash University Werribee Animal Facility under conditions approved by the Monash University Animal Ethics Committee (AEC SOBSA/P/2009/98). Sheep were provided with water and standard feed ad lib and fasted 16 h before oral immunisation. Sheep were randomly assigned into four groups of 2–5 animals each (Table 1). A single sheep from the transgenic rLTB expressing leaf vaccine group (LTB-Leaf) developed balanopsthitis (pizzle rot) 14 days after beginning the trial and was treated with a testosterone implant. This sheep was not excluded from analyses.

Sheep were immunised on days 0, 14 and 28 followed by a boost dose on day 38, four days before sacrifice. Vaccine materials were formulated immediately before delivery by mixing 19 g freeze-dried plant material with 200 ml of an oil based emulsion (125 ml peanut oil:75 ml dH_2_O). When receiving the transgenic rLTB plant-based vaccines (LTB-HR or LTB-Leaf), each dose was sufficient to deliver 5 mg rLTB. Sheep receiving the CtHR or CtLeaf vaccines were immunised with the equivalent volume of formulated control plant materials. The formulated vaccines were administered orally to sheep by gavage directly into the rumen to simulate drenching, a common delivery system used routinely to worm domestic sheep flocks. At trial termination (day 42), sheep were humanely killed by intravenous injection with a lethal dose of lethobabarb (100 mg/kg bodyweight).

**Table 1:** Oral immunisation treatments and number of sheep assigned to each group.

Treatment	Sheep
Control hairy root (CtHR)	3 (Sheep #50, 28, 54)
Control leaf (CtLeaf)	2 (Sheep #37, 73)
Transgenic hairy root containing 5mg rLTB (LTB-HR)	5 (Sheep #29, 30, 31, 42, 75)
Transgenic leaf containing 5mg rLTB (LTB-Leaf)	5 (Sheep #36, 47, 57, 64, 69)

### Collection and processing of biological specimens

#### Serum collection

Blood samples were taken from the jugular vein using an 18 G needle immediately before the first immunisation (pre-immune), 14 days after each of the first three doses and four days (at trial termination) after the boost. The blood was clotted at room temperature (20–22°C) overnight and serum separated by centrifugation at 400 *g* for 10 min and stored at −20°C until required for LTB-specific antibody detection by ELISA.

#### Sampling and in vitro culture of mesenteric lymph nodes

At post-mortem, four lymph nodes were taken from the mesentery, the first at the abomasum/duodenum junction (MLN 1) and the next three along the first 0.5 m of the small intestine (MLN 2–4). MLNs were subjected to an antigen-specific antibody secreting cell (ASC) assay for detection of LTB-specific antibody responses using a protocol modified from those previously described [23], [24]. MLNs were dissected into small pieces and cultured in 24-well flat-bottomed tissue culture plates. One MLN and 1 ml complete DMEM medium (Gibco) containing 10% (v/v) heat-inactivated foetal calf serum (Thermo), 100 U/ml penicillin (Gibco), 0.1 mg/ml streptomycin (Gibco) and 2 mM glutamine (Gibco) per well were incubated at 37°C in a humidified incubator with 5% CO_2_ for 24 h. Culture supernatants (ASC supernatants) were collected and stored at −20°C and the presence of LTB-specific antibodies determined by ELISA.

#### Sampling the mucosa of the abomasum

The mucosal lining of the abomasum was sampled by scraping the inside surface with a glass slide. Mucus scrapings were prepared for ELISA as described by [25]. Abomasal scrapings were washed off the slide into a 50 ml tube with 3 ml PBST supplemented with 2x Roche Complete Protease Inhibitor Cocktail tablets (PBST2I). The supernatant was collected following centrifugation at 9000 *g* for 15 min at 4°C and stored at −20°C until required.

#### Small intestine washes to sample intestinal secretions

Four sections of the small intestine were excised, each section measured 0.5 m in length and was taken 3 m apart, beginning at the abomasum/duodenum junction (section 1, 0–0.5 m). Sections 2–4 were sampled at 3.5–4 m, 7–7.5 m and 10.5–11 m respectively. Each segment was flushed with 20 ml saline then incubated for 30 min with 10 ml saline and gentle rocking. Each end of the intestinal segments was clamped during washes to prevent leakage. Washes containing intestinal secretions were collected and stored at −20°C until required.

#### Faecal sampling

Faecal samples were collected before vaccination on day 0 and again at day 16 and 36 h after immunisation with the second oral dose allowing administered vaccine material to complete transit through the sheep GIT [26]. Faecal matter was homogenised in 1 ml/g PBST2I with two 3 mm tungsten carbide beads for 1 min at a frequency of 28/s in a Qiagen Mixer Mill. The homogenate was cleared by centrifugation at 13,000 rpm at 4°C for 10 min and capture ELISA performed (as described above) to detect and quantify LTB in the supernatant.

### ELISA to determine LTB-specific IgG and IgA antibody titre

LTB-specific ELISA was used to assess IgG and IgA antibody responses in immunised sheep. Costar 9018 96-well microtitre plates (Corning Life Sciences) were coated with 50 µl/well chicken CTB antibody (Sigma-Aldrich) diluted 1∶5,000 in PBS. Plates were sealed and incubated at 4°C overnight. Three washes with PBST were performed following each incubation. Plates were blocked with 5% DM in PBST at 37°C for 1 h before a further incubation for 1 h at 37°C with 0.25 µg/ml *Pichia*-made rLTB (Sigma-Aldrich). Serial dilutions of the various biological samples were made on the plate with starting dilutions in PBST as follows – 1∶1000 or undiluted serum for IgG or IgA determination respectively, 1∶2 ASC supernatant, 1∶4 small intestine wash and undiluted abomasum mucus. Plates were incubated overnight at 4°C before incubation with 1∶2,000 mouse anti-sheep/goat IgG HRP conjugate (Enzo Life Sciences), or rabbit anti-sheep IgA HRP conjugate (Novus Biologicals) at 37°C for 2 h. Bound LTB-specific antibodies were visualised using TMB-peroxidase substrate (Bio-Rad Laboratories) according to manufacturer's instructions. Endpoint antibody tire was reported as the highest dilution with an absorbance of four standard deviation units above background (mean absorbance of at least three wells lacking biological sample). All measurements were performed in triplicate, the geometric mean titre determined and data subjected to statistical analysis using the non-parametic one way analysis of variance (ANOVA) and student's t-test. Data from sheep receiving control vaccine treatments (CtHR and CtLeaf) were combined for analysis. An antigen-specific antibody response exceeding the geometric mean titre of the control group (background) by at least three standard deviations was considered a positive response.

## Results

### Plant Materials

Two different plant species *N. benthamiana* and *Petunia parodii* (petunia) were chosen due to their lack of use in the animal or human food chain to reduce the chance of contamination of the food chain and due to their ease of transformation. Although this resulted in more than one variable in the study our previous study demonstrated their optimal nature for oral delivery to mice [3] and we hence sought to delineate their ability to orally deliver to ruminants.

### LTB-specific antibody responses in serum

**Figure 1:**
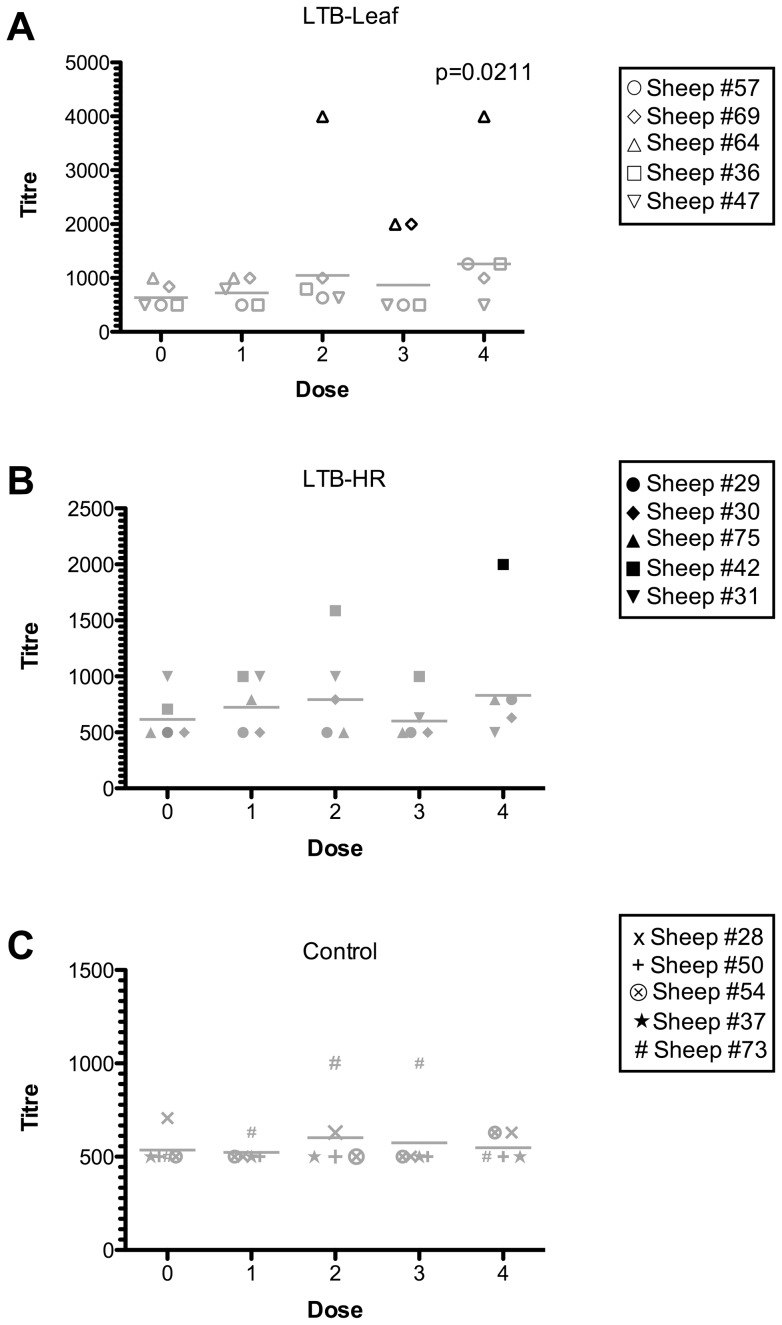
LTB-specific IgG antibody titres in serum collected from sheep before immunisation with LTB-Leaf (A), LTB-HR (B) or control vaccines (C). Black symbols denote positive responders defined as sheep with antibody titres at least three standard deviations above the control mean, non-responders are indicated by grey symbols. The horizontal lines represent geometric means, statistical analysis (Student's t-test determined a significant difference between the means of the control and LTB-Leaf groups after four doses, p<0.05).

Immunisation of sheep with the LTB-Leaf vaccine resulted in a higher number of sheep with positive antigen-specific IgG-serum responses than those receiving the LTB-HR vaccine (Fig. 1). The mean titre observed for sheep immunised with the LTB-Leaf vaccine was significantly different from controls after four vaccine doses. In one of the five LTB-Leaf immunised sheep (Sheep #64), the maximum IgG-serum response was observed after two immunisations (Fig. 1A) and was not increased by an additional two doses. After three doses, the number of reactive LTB-Leaf immunised sheep was doubled, but this response waned in one animal (Sheep #69) by trial's end. In contrast, four doses of the LTB-HR vaccine were required to produce a single animal (Sheep #42) with reactive serum (Fig. 1B). LTB-specific IgA antibodies were not detected in sera, irrespective of the vaccine or number of doses administered. The baseline antibody titres observed in pre-immune serum could be attributed to a low level of *E. coli* colonisation in animals, which were not housed in germ-free conditions.

### LTB-specific antibody responses in antibody secreting cells of mesenteric lymph nodes

**Figure 2:**
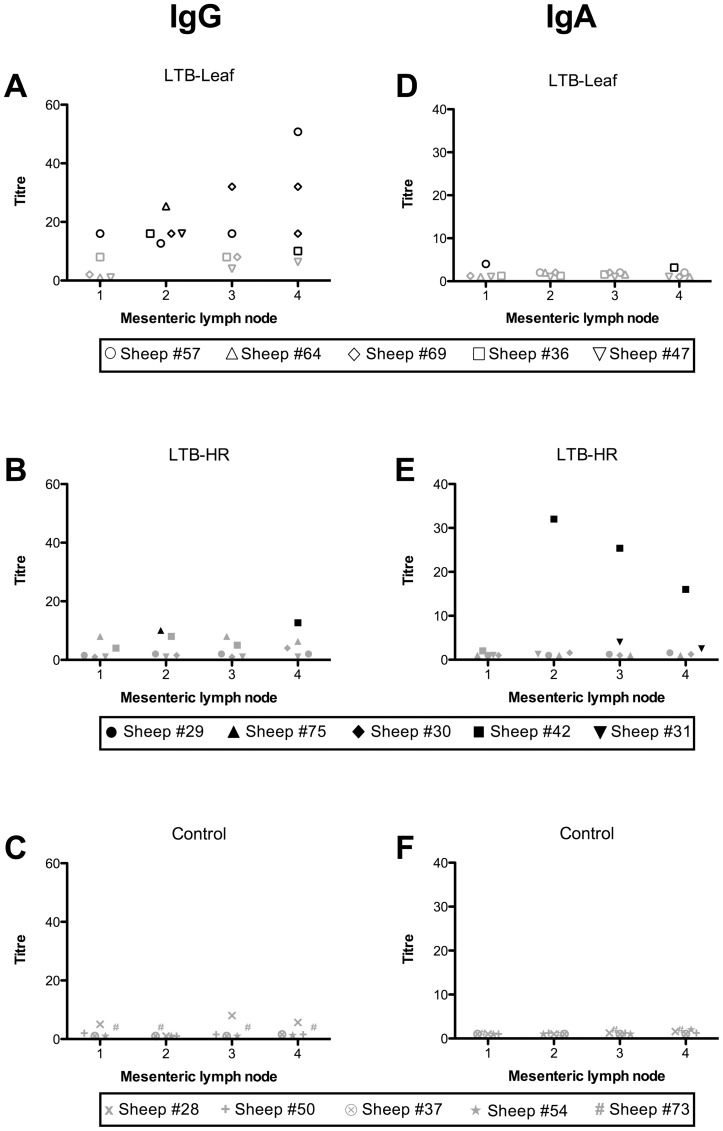
LTB-specific antibody titres in MLNs collected from successive sites along the small intestine of the sheep GIT following oral immunisation with four doses of LTB-Leaf (A and D, IgG and IgA respectively), LTB-HR (B and E, IgG and IgA respectively) or control (C and F, IgG and IgA respectively) plant materials. MLN 1 was sampled from the abomasum/duodenum junction, MLN 2–4 were the next three lymph nodes sampled from the first 0.5 m of the small intestine. Black symbols denote positive responders defined as sheep with antibody titres at least three standard deviations above the control mean, non-responders are indicated by grey symbols.

Detection of antibody production in serum following oral immunisation may not be indicative of immune responses at mucosal sites [24]. The ASC assay was adopted as a potentially more sensitive method for detection of antigen-specific antibody production from MLNs draining the intestinal tissue. Unlike the serum analysis, both IgG and IgA antibody isotypes were detected in MLN-derived ASC supernatants taken from LTB-HR or LTB-Leaf immunised sheep (Fig. 2).

All five sheep immunised with the LTB-Leaf vaccine assayed positive for an LTB-specific ASC-IgG response at one or more of the MLN sites sampled (Fig. 2A). One sheep from the LTB-Leaf group (Sheep #57) exhibited a positive ASC-IgG response at all four MLNs. This same sheep, along with Sheep #36 were also positive for an ASC-IgA response at MLNs 1 and 2 respectively (Fig. 2D). Of the LTB-HR immunised sheep, Sheep #42 and 31 displayed at least one positive ASC response for both IgG and IgA isotypes with maximum IgA titres recorded for Sheep #42 at three MLN sites (Fig. 2E).

### LTB-specific antibody responses in the abomasal mucosa and secretions of the small intestine

**Figure 3:**
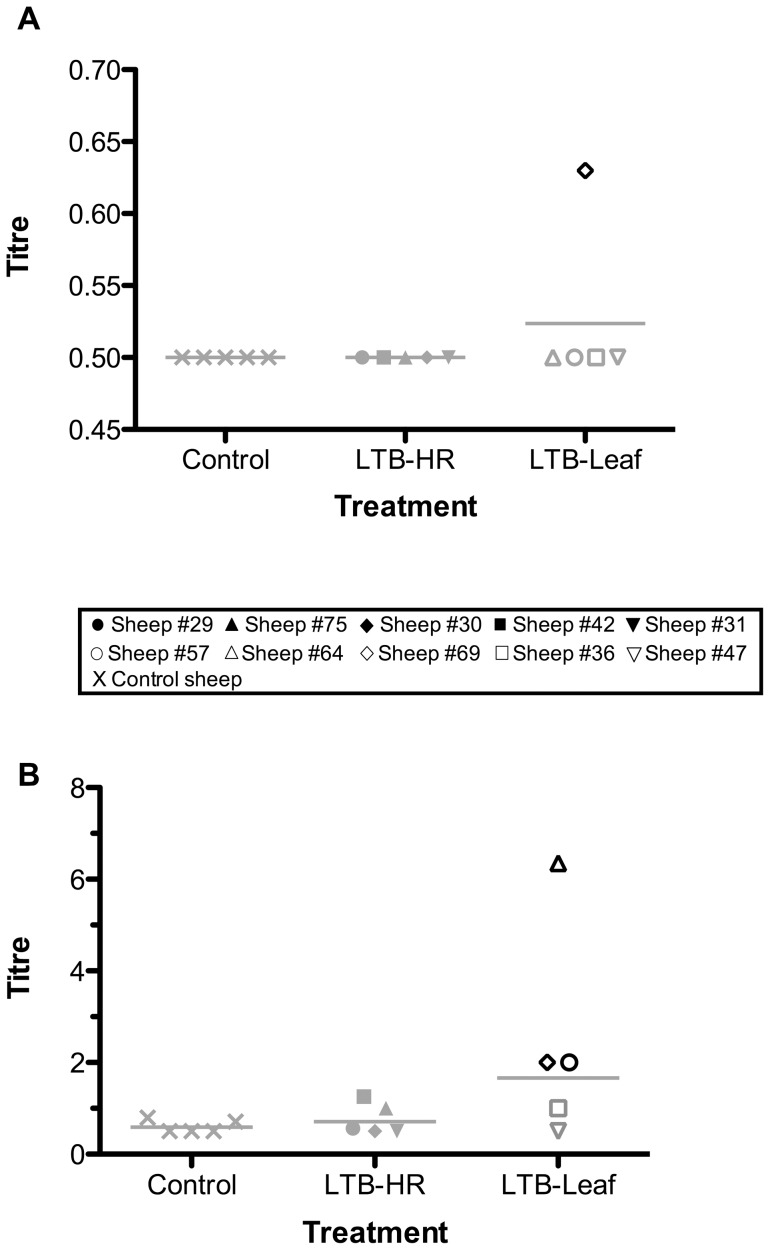
LTB-specific IgG (A) and IgA (B) antibody titres in abomasum mucus following oral immunisation with four doses of control or LTB-transgenic plant materials. The horizontal lines represent geometric means. Black symbols denote positive responders defined as sheep with antibody titres at least three standard deviations above the control mean, non-responders are indicated by grey symbols.

Induction of LTB-specific antibody responses in the mucosa of the abomasum was identified only after immunisation with the LTB-Leaf vaccine (Fig. 3). At this site three sheep were identified as positive responders with IgA titres above those observed for the control group (Fig. 3B). One of these sheep (Sheep #69) also exhibited an elevated IgG titre (Fig. 3A).

**Figure 4:**
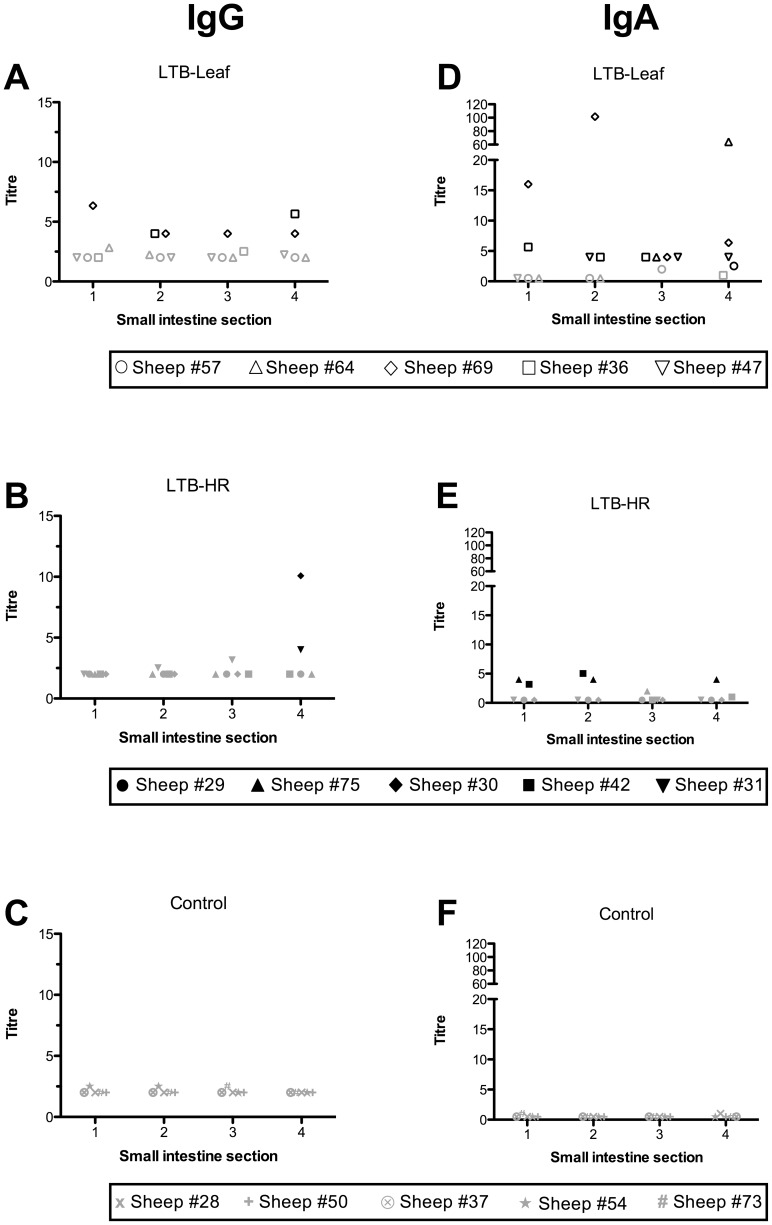
LTB-specific antibody titres detected in intestinal washes performed at four sites along the first 11 **m of the sheep small intestine following oral immunisation with four doses of LTB-Leaf (A and D, IgG and IgA respectively), LTB-HR (B and E, IgG and IgA respectively) or control (C and F, IgG and IgA respectively).** Sections 1, 2, 3 and 4 are defined as the first 0–0.5 m, 3.5–4 m, 7–7.5 m and 10.5–11 m respectively from the abomasum/duodenum junction. Black symbols denote positive responders defined as sheep with antibody titres at least three standard deviations above the control mean, non-responders are indicated by grey symbols.

**Figure 5:**
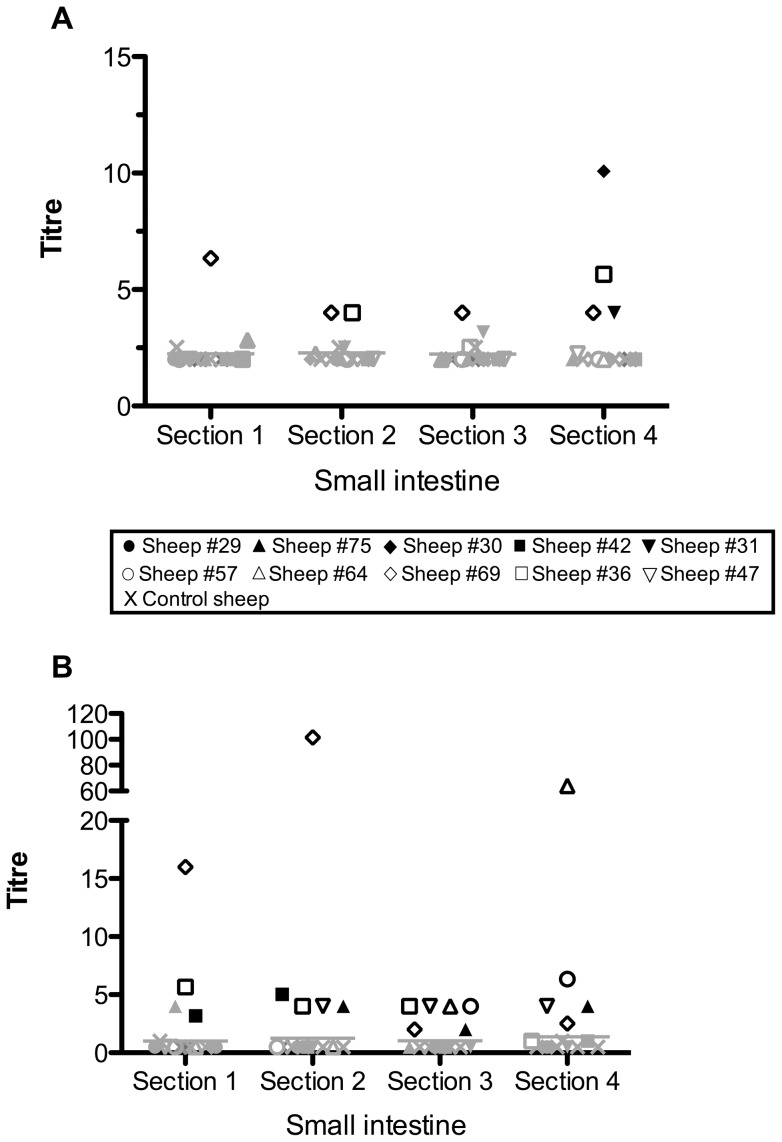
Relative abundance of LTB-specific IgG (A) and IgA (B) at different sections of the sheep small intestine following oral immunisation with four doses of control or LTB-transgenic plant materials. The horizontal lines represent geometric means. Black symbols denote positive responders defined as sheep with antibody titres at least three standard deviations above the control mean, non-responders are indicated by grey symbols.

LTB-specific IgG antibody was detected in intestinal washes of two of the five sheep immunised with the LTB-Leaf vaccine (Fig. 4A). In one of these sheep (Sheep #69) the response was detected at all four sections sampled from the small intestine (Fig. 4A). The number of antigen-specific IgG positive LTB-Leaf immunised sheep increased from one to two when washes were taken at sections 2 and 4 (3.5–4 m and 10.5–11 m respectively) of the small intestine (Fig. 4A). It was at the most distant site sampled that two IgG positive LTB-HR immunised sheep were also identified (Fig. 4B). All sheep immunised with the LTB-Leaf vaccine also exhibited a positive IgA response at one or more sites sampled along the small intestine (Fig. 4D). LTB-specific IgA responses in the small intestine were stimulated above controls in two LTB-HR immunised sheep at all sections except section 3 (7–7.5 m; Fig. 4E); one of these sheep (Sheep #75,) was also positive at section 4 (10.5–11 m; Fig. 4E). Of the sites sampled along the small intestine, the most immunologically responsive with respect to immunoglobulin production was section 4 (10.5–11 m) for IgG (Fig. 5A), whilst IgA was more widespread, observed at sections 2 to 4 (3.5–11 m; Fig. 5B).

### Detection of LTB in faeces

Faecal samples were assayed for LTB to determine whether the vaccine plant materials had resisted breakdown during passage through the sheep GIT. LTB was not detected in faecal samples taken from pre- and post-immune sheep from control, LTB-HR or LTB-Leaf groups (data not shown).

## Discussion

The pharmaceutical industry is constantly assessing methods for improved delivery for vaccines, pharmaceuticals and nutraceuticals. The oral route increases ease of delivery, is less expensive, and encourages increased compliance by eliminating the need for needles. Moreover, oral delivery is particularly desired for immunising free-ranging domestic animals that are typically ruminants. Numerous studies have reported immunogenicity of orally delivered plant-made vaccines in humans and small animal models, but few have demonstrated their efficacy in ruminants [27], [28], [29], [30]. We have previously determined that the way plant-made vaccine material is delivered influences immunological outcomes in mice [3]. We therefore now investigate how plant-made vaccine material delivery influences immunological outcomes in sheep, an important end user ruminant and also a model for other ruminants such as goat and cattle.

LTB was chosen as our model antigen because it can be produced in a wide variety of plant systems [3], [16], [19], [20], is stable under acidic conditions [31] and in the GIT [15] and has immunogenic properties when delivered orally. Its affinity for binding the GM1 receptor to mediate transepithelial flux from the lumen into the abluminal environment also makes LTB a potentially important component as an immune modulator in the design of subunit vaccines. Similarly, the plant system used to orally deliver a vaccine candidate merits careful consideration. Destruction of pH-sensitive antigens in the acidic environment of the sheep abomasum could be avoided if delivered from a root-based vaccine to manipulate release into the small intestine. In the present study, mucosal (abomasal, intestinal and ASC-derived IgA and IgG) and systemic (serum IgG) immune responses were achieved in sheep orally immunised with plant-made LTB vaccines delivered from root and leaf material. Local antibody detection at mucosal sites was more sensitive than serum. Of the LTB-HR and LTB-Leaf vaccines delivered, the latter stimulated more robust antigen-specific antibody responses at mucosal sites of the GIT, including the stomach and small intestine, in serum and MLNs.

Vaccine materials were formulated in oil and administered directly into the rumen of the sheep via a tube inserted down the oesophagus. The delivered plant materials were sieved to achieve a uniform particle size of 0.5–1 mm^2^ to better protect the antigen from degradation by minimising the time spent in the rumen. In sheep, particles with diameters larger than 1.18 mm transit through the rumen slower than smaller particles [32]; this has also been found in cattle with increased forage particle size improving fibre digestibility by increasing retention time in the rumen [33].

From the rumen, the vaccine transits through the reticulum and omasum before reaching the abomasum (true stomach) where enzymatic digestion of protein, carbohydrates and lipids is initiated. It is anticipated that breakdown of the plant cells encapsulating the rLTB antigen begins in the rumen and continues in the reticulum, the principal sites for cellulose digestion in ruminant species. It was in the abomasum mucus that antibody responses were first observed following administration of the LTB-Leaf vaccine. This suggests that as the leaf material begins to degrade the antigen remains sufficiently protected during rumination, presumably by the lipid coating provided by the oil formulation matrix. In contrast, the lack of antibody response in abomasum mucus from the LTB-HR vaccine suggests that root tissue may be comparatively more resistant to rumination and enzymatic digestion resulting in delayed antigen release.

Although GALT is absent in the abomasum, immune responses can be induced when the mucosal epithelium is penetrated [2]. LTB is particularly efficient in crossing the epithelium from the lumen primarily via binding to ganglioside GM1 along with other mammalian galactoglycoprotein receptors [13], [14]. Moreover, direct sampling of antigen from the mucosal lumen may also occur via intra- and sub-epithelial DCs [2], [34]. Once the antigen has traversed the mucosal epithelium it is transported by DCs via the lymphatics to draining MLNs where antigen-specific B cells are generated and then returned to mucosal sites via the blood stream [2], [35].

From the abomasum, the vaccine materials enter the small intestine. By this stage breakdown of the plant cells and formulation matrix should be completed, releasing the remainder of its antigenic cargo. It was in the small intestine that the most robust mucosal immune responses were detected from both the LTB-Leaf and LTB-HR vaccines, the leaf material producing elevated IgA titres compared to other treatments in all five sheep receiving this vaccine. It was of interest that section 4, the section further through the GIT, was the site where the most robust antigen-specific IgG responses were found while IgA responses expanded to earlier sites (sections 2 to 4). The consistency in the immune response observed at the small intestine, particularly for the LTB-Leaf group, is noteworthy given the potential for variable responses when using an outbred sample of sheep.

LTB-specific IgA antibodies were absent in all sera, irrespective of vaccine treatment or number of doses administered. This is not unexpected as detection of antibody production in serum following mucosal immunisation can be typically difficult particularly when responses are low [24]. An alternative approach, previously validated in several studies, was utilised to detect antibodies secreted by MLNs using the ASC assay [23], [36]. Elevated IgA titres were detected in the MLNs of two LTB-Leaf- and LTB-HR-vaccinated sheep as compared to other treatments. In addition, MLN 2 was identified as the most active site for generating an IgG response with all LTB-Leaf- (two more than that identified from serum) and one LTB-HR-vaccinated sheep exhibiting stimulated titres. It is interesting to note that the different plant vehicles induced different isotype responses at the MLNs with root-delivered LTB elevating IgA titres in contrast to the stimulated IgG titres observed for the leaf-delivered counterpart.

Whilst most of the immune inductive sites of the GIT are located in the GALT of the small intestine, the potency of the LTB-Leaf vaccine benefitted from an early release in the abomasum perhaps due to the stability of LTB and the resulting prolonged antigen exposure at mucosal surfaces and priming distal sites in the small intestine. Antibody responses at the tonsils or other lymphoid tissues of the oral and nasopharyngeal cavities were not sampled in this study but should not be discounted as additional sites within the mucosal epithelium that could be exploited for induction of immune responses from plant-made vaccines. Plant material in its nature is fibrous and as such is often regurgitated from the rumen during fermentation for further mechanical breakdown by chewing and can result in repeated and sustained exposure of the plant-delivered antigen to the tonsils priming more distal sites of the GIT or respiratory system [28].

It is apparent that both the leaf- and root-based vaccine preparations protected the antigenic load sufficiently during rumination and enzymatic digestion to enable its delivery to relevant immune responsive sites. Furthermore, the type of plant tissue used can manipulate timing of antigen release. In our experience, antigen release from both leaf- and root-based vaccines has been consistent across sheep (present study) and mouse [3] animal models. In each case the leaf-based vaccine facilitated early antigen release in the true stomach of orally immunised sheep and mice, whilst the root-based vaccine delayed release to the small intestine. Improved antigen release and antibody responses from root-based vaccine delivery vehicles may be served by different plant species, altered culture conditions or harvest times.

The plant material used to deliver LTB orally to sheep affected immunogenicity. This finding suggests that a delicate balance between protecting the vaccine antigen against digestive degradation and enabling release for presentation of the antigen at immune responsive sites needs to be struck to maximise vaccine efficacy. Although *N. benthamiana* leaf material provided the optimal oral delivery vehicle for induction of mucosal immune responses to LTB in both monogastric (mouse) and ruminant (sheep) models, it is anticipated that plant choice will need to be assessed on a case by case basis, taking into account antigen stability.

Optimising oral delivery of plant-made, valuable proteins will have broad ramifications to animal as well as human health. Oral delivery will facilitate treatment of free-ranging domesticated and native animal populations that may otherwise go untreated, broaden opportunities for existing pharmaceuticals and create opportunities for new compounds and target populations.
